# Circulating plasma factors induce tubular and glomerular alterations in septic burns patients

**DOI:** 10.1186/cc6848

**Published:** 2008-03-25

**Authors:** Filippo Mariano, Vincenzo Cantaluppi, Maurizio Stella, Giuseppe Mauriello Romanazzi, Barbara Assenzio, Monica Cairo, Luigi Biancone, Giorgio Triolo, V Marco Ranieri, Giovanni Camussi

**Affiliations:** 1 Dipartimento di Area Medica, Unita' di Nefrologia e Dialisi, Ospedale CTO, Via G. Zuretti 29, Torino, 10126, Italy; 2 Dipartimento di Medicina Interna, Centro Interdipartimentale di Biotecnologie Molecolari e Centro Ricerca Medicina Sperimentale (CeRMS), Universita' di Torino, Corso Dogliotti, 14, Torino, 10126, Italy; 3 Dipartimento di Chirurgia Plastica, Centro Grandi Ustionati, Ospedale CTO, Via G. Zuretti 29, Torino, 10126, Italy; 4 Dipartimento di Anestesiologia e Rianimazione, Università di Torino, Ospedale S Giovanni Battista-Molinette, Corso Dogliotti 14, Torino, 10126, Italy

## Abstract

**Background:**

Severe burn is a systemic illness often complicated by sepsis. Kidney is one of the organs invariably affected, and proteinuria is a constant clinical finding. We studied the relationships between proteinuria and patient outcome, severity of renal dysfunction and systemic inflammatory state in burns patients who developed sepsis-associated acute renal failure (ARF). We then tested the hypothesis that plasma in these patients induces apoptosis and functional alterations that could account for proteinuria and severity of renal dysfunction in tubular cells and podocytes.

**Methods:**

We studied the correlation between proteinuria and indexes of systemic inflammation or renal function prospectively in 19 severe burns patients with septic shock and ARF, and we evaluated the effect of plasma on apoptosis, polarity and functional alterations in cultured human tubular cells and podocytes. As controls, we collected plasma from 10 burns patients with septic shock but without ARF, 10 burns patients with septic shock and ARF, 10 non-burns patients with septic shock without ARF, 10 chronic uremic patients and 10 healthy volunteers.

**Results:**

Septic burns patients with ARF presented a severe proteinuria that correlated to outcome, glomerular (creatinine/urea clearance) and tubular (fractional excretion of sodium and potassium) functional impairment and systemic inflammation (white blood cell (WBC) and platelet counts). Plasma from these patients induced a pro-apoptotic effect in tubular cells and podocytes that correlated with the extent of proteinuria. Plasma-induced apoptosis was significantly higher in septic severe burns patients with ARF with respect to those without ARF or with septic shock without burns. Moreover, plasma from septic burns patients induced an alteration of polarity in tubular cells, as well as reduced expression of the tight junction protein ZO-1 and of the endocytic receptor megalin. In podocytes, plasma from septic burns patients increased permeability to albumin and decreased the expression of the slit diaphragm protein nephrin.

**Conclusion:**

Plasma from burns patients with sepsis-associated ARF contains factors that affect the function and survival of tubular cells and podocytes. These factors are likely to be involved in the pathogenesis of acute tubular injury and proteinuria, which is a negative prognostic factor and an index of renal involvement in the systemic inflammatory reaction.

## Introduction

Acute renal failure (ARF) is frequently associated with a systemic inflammatory response due to sepsis [1]. Circulating factors have been proposed as responsible mediators for the systemic micro-vascular injury occurring in these patients [2]. During sepsis, bacterial endo- or exotoxins can act on glomerular podocytes and tubular epithelium, stimulating synthesis of cytokines and other inflammatory mediators [3-6]. In patients with sepsis-associated ARF, a marked dissociation between the degree of tubular necrosis and the renal dysfunction has been described [7]. By contrast, clinical and experimental data suggest that apoptosis plays an important role in sepsis-induced ARF [4-9].

In severe burns patients, sepsis almost always develops, lasts for several weeks and is frequently associated with ARF [10-19]. A constant feature of burn-associated kidney injury is proteinuria, which starts in the first days post-injury and increases over time. Proteinuria is a consequence of increased glomerular permeability and of decreased tubular re-absorption of filtered proteins [13,20,21].

Immediately after burn injury, onset of proteinuria can depend on filtered breakdown proteins derived from massive tissue destruction or on renal involvement as a consequence of the increased systemic capillary permeability. Subsequently, proteinuria reflects the involvement of the kidney in the septic process [13,20-22].

In the present study, we investigated whether circulating factors present in the plasma of septic severe burns patients could induce tubular and glomerular alterations that could account for proteinuria and ARF.

## Materials and methods

### Patients

From January 2003 to December 2005, from 258 patients admitted to the Burn Center (Dipartimento di Chirurgia Plastica, Centro Grandi Ustionati, Ospedale CTO, Via G. Zuretti 29, Torino, 10126, Italy) (mean (standard error (SE) burned surface area 24.5 ± 1.3%, range 1–98%, mortality rate 19.4%), 19 patients with severe burns and septic shock who developed ARF (8–10 days after burn injury) were enrolled in a prospective study ("burns septic ARF" group). Demographic and clinical data (Table 1) for these 19 patients were recorded and blood and urine biochemical parameters were analyzed. Plasma samples collected at the time of ARF onset before the start of renal replacement therapy (RRT) were used for laboratory studies. Informed consent was obtained according to the Declaration of Helsinki and study approval was obtained by the Center for Molecular Biotechnology Institutional Review Board, University of Torino.

**Table 1:** Demographic and clinical characteristics

	Septic burns patients with ARF (n = 19)	Septic burns patients (n = 10)	Septic ARF patients (n = 10)	Septic patients (n = 10)
Female (n)	7 (36.8%)	2 (20%)	4 (40%)	3 (30%)
Age (years)	50.4 ± 4.6	55.5 ± 6.0	62.4 ± 4.4	58.2 ± 3.8
Burned surface area (%)	51.3 ± 5.4	44.9 ± 6.2	-	-
SOFA score (at ARF onset)	11.8 ± 0.4	-	11.7 ± 1.1	-
Non-survivors (n)	10 (52.6%)	8 (80%)	8 (80%)	5 (50%)
Renal replacement therapy (n)	15 (78.9%)	-	10 (100%)	-
Duration of renal replacement therapy (days)	22.4 ± 2.6	-	12.9 ± 2.7	-
				
Hemoculture*	n (all patients)			
*Staphylococcus aureus*	12			
*Acinetobacter baumannii*	11			
*Pseudomonas aeruginosa*	9			
*Candida albicans*	4			
*Escherichia coli*	1			
*Klebsiella pneumoniae*	1			

*All patients had positive blood cultures. Some patients were simultaneously positive for more than one pathogen strain.

Data shows mean ± standard error (SE) unless otherwise stated.

ARF, acute renal failure; SOFA, Sequential Organ Failure Assessment.

All 19 patients fulfilled the criteria for septic shock [23] and ARF according to Acute Dialysis Quality Initiative – Risk, Injury, Failure, Loss, and End-stage kidney disease (ADQI-RIFLE) classification [24]. Patients with chronic cardiovascular system failure (New York Heart Association (NYHA) class III), chronic respiratory failure (chronic hypoxia, hypercapnia), chronic liver failure (biopsy-confirmed cirrhosis or portal hypertension), neoplastic diseases, collagenopathies, insulin-dependent diabetes mellitus and known aortic aneurysm were excluded from the study.

As controls, we studied 10 burns patients with septic shock without ARF ("burns septic" group), 10 septic shock patients with ARF ("septic ARF" group), 10 patients with septic shock without ARF ("septic" group), 10 stable uremic patients ("uremic" group) and 10 healthy volunteers ("healthy" group) (Table 1).

Systemic treatment of burns patients was based on current guidelines and consisted of prompt hydro-electrolytic replacement, ventilatory support when necessary, pain control and surgical treatment by multiple stage skin excisions and subsequent reconstruction by means of autografts or donor allografts assured by the Regional Skin Bank. Septic events mainly caused by multiresistant bacterial strains were treated according to current guidelines with antibiotic regimens following blood cultures and sensitivity tests. When glycopeptides or aminoglycosydes were used, the administered dose was calculated on creatinine clearance and adjusted following the results of drug blood levels [10].

### Biochemical parameters

The clinical observation of the burns septic ARF group was divided in two periods; the first from burn injury to the onset of ARF (pre-ARF period) and the second from the onset of ARF to functional recovery or to exitus (ARF period). Recovery from ARF was defined as blood creatinine improvement back up to starting values. The mean times of observation in pre-ARF and ARF periods were 18.9 ± 3.6 and 19.8 ± 3.8 days, respectively (mean ± SE).

Biochemical evaluation included blood creatinine clearance (BCrC) and blood urea clearance (BUC) measured by 24-h diuresis, fractional excretion of sodium (FeNa) and potassium (FeK), and hemochromocytometer examination.

Proteinuria was determined in an automated analyzer by benzethonium chloride dye binding method (Roche Modular System, Roche Diagnostics GmbH, Mannheim, Germany) [25].

Tumor necrosis factor (TNF)α concentrations in plasma were measured by Bio-Plex cytokine assay (Biorad, Hercules, CA, USA).

### Laboratory studies

#### Cells

Podocytes and proximal tubular epithelial primary cultures were obtained from normal cortex fragments of surgically removed kidneys. Immortalized tubular cells and podocyte lines were generated by infection with a hybrid Adeno5/SV40 virus as previously described [26,27].

#### Viability and apoptosis assays

Cellular viability was studied by using the XTT-based colorimetric method (Sigma, St Louis, MO, USA).

In all groups studied, apoptosis was evaluated by terminal uridine deoxynucleotidyl transferase dUTP nick-end labeling (TUNEL) assay (ApopTag, Oncor, Gaithersburg, MD, USA). Moreover, in selected experiments tubular apoptosis was confirmed by identification of intranucleosomal DNA fragmentation after 1% agarose gel electrophoresis (BioVision Research Products, Mountain View, CA, USA). The activities of caspases 3, 8 and 9 were assessed by a colorimetric assay (Chemicon International, Temecula, CA, USA).

#### Polarity assay

Transepithelial electrical resistance (TER), an indicator of cell polarity, was measured by using an epithelial volt-ohm meter (EVOM, World Precision Instruments, Inc., Sarasota, FL, USA) after incubation of tubular cells or podocytes with different stimuli. All measures were normalized for the area of the membrane used in the experimental procedure and expressed as ohm/cm^2^.

#### Permeability assay

Permeability was evaluated by diffusion of Trypan blue-albumin complexes across podocyte confluent monolayers cultured on Transwell (Greiner Bio-One, Frickenhausen, Germany) under conditions of continuous slight agitation. Aliquots of medium from the upper and the lower wells were transferred to a 96-well plate and analyzed at the 590 nm wavelength (Model 680 Spectrophotometer, Biorad, Hercules, CA). Results are given as percentage of increase of albumin diffusion in comparison to vehicle alone [28].

#### Gene array

The Human GEarray for the study of apoptosis markers (Superarray Inc., Bethesda, MD, USA) was performed and analyzed according to the manufacturer's instructionsto characterize the expression profile of tubular cells incubated in presence of different plasma samples. These data are freely available from the ArrayExpress databank of the European Bioinformatics Institute (experiment name: Human Apoptosis – Burn; ArrayExpress accession: E-MEXP-1510).

#### Immunofluorescence, fluorescence-activated cell sorting (FACS) and Western blot analysis

For immunofluorescence studies, antibodies directed against Fas (CD95), CD40 (Upstate, Charlottesville, VA, USA), megalin, ZO-1, E-cadherin and pan-cytokeratins (Santa Cruz Biotech, Santa Cruz, CA, USA) were used with tubular cells. Fas and CD40 expression were also evaluated by FACS analysis (Becton Dickinson, Mountain View, CA, USA). Antibodies directed against nephrin (Progen, Heidelberg, Germany), nestin (Santa Cruz) and ZO-1 were used with podocytes. Specific Alexa Fluor-conjugated antibodies were used as secondary antibodies (Invitrogen, Carlsbad, CA, USA). Fluorescein isothiocyanate (FITC)-conjugated phalloidin (Sigma) was used to evaluate cytoskeleton actin distribution on tubular cells and podocytes by ultraviolet (UV) light microscopy.

For Western blot analysis, cell lysates were separated by SDS-PAGE and immunoblotted with anti-human Bax, anti-human Bcl-2, anti-human megalin (tubular cells) or anti-human nephrin (podocytes) antibodies.

### Statistical analysis

Descriptive statistics, Student t test, linear regression analysis with curves of minimal square and analysis of variance (ANOVA) with Dunnet or Newman-Keuls multi-comparison test (Statistica 6.1, StaSoft Inc, Tulsa, OK, USA) were performed. Values were expressed as mean ± SE. p Values < 0.05 were considered statistically significant.

## Results

### Proteinuria was related to patient outcome and systemic inflammation

**Figure 1:**
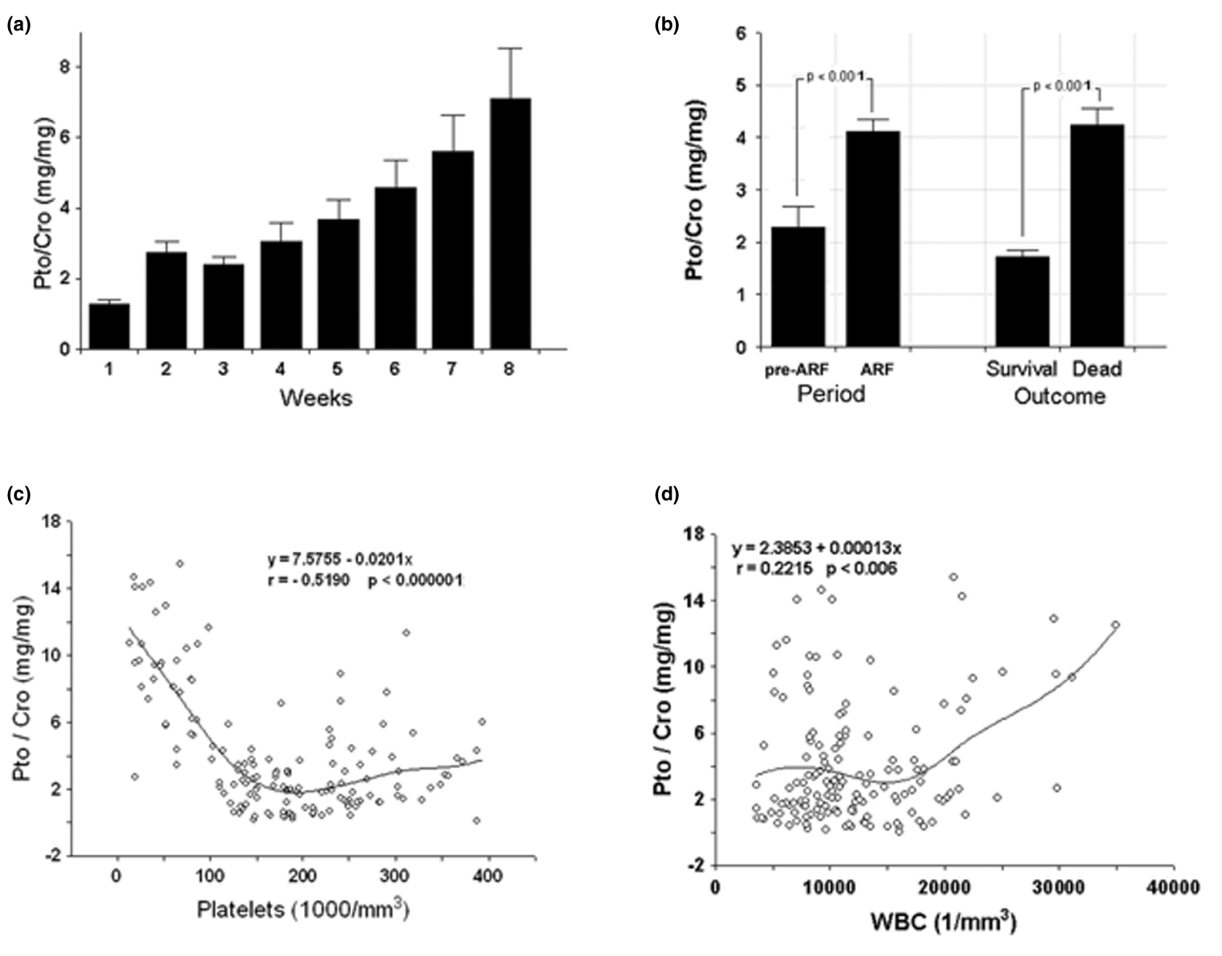
Proteinuria correlates with patient outcome and with markers of systemic inflammation. **(a) **Proteinuria expressed as proteinuria/creatininuria ratio (Pto/Cro, mg/mg) in the weeks following patient admission. Data are given as weekly average of daily values. Only non-oliguric patients were included and the number of patients for each week was: week 1, n = 19; week 2, n = 19; week 3, n = 17; week 4, n = 15; week 5, n = 10; week 6, n = 7; week 7, n = 6; week 8, n = 5. **(b) **Overall mean proteinuria in pre-acute renal failure (ARF) vs ARF periods and in deceased vs surviving patients. **(c, d) **Relationship between Pto/Cro and indexes of systemic inflammatory state in ARF period. Pto/Cro negatively correlated with platelet count **(c) **and positively with white blood cell (WBC) count **(d)**. Student t test and linear regression analysis were performed where appropriate. Data for different parameters are also shown as minimal square fitting curves.

Clinical characteristics of the 19 burns septic ARF group patients with positive blood cultures are given in Table 1. The burns septic ARF patients presented marked and persistent proteinuria (mean ± SE value during 8-week observation period of 2,074.8 ± 113.4 mg/day). When proteinuria was expressed as proteinuria/creatininuria ratio (Pto/Cro), a continuous increase of protein excretion was observed over the time (Figure 1A). In addition, Pto/Cro was significantly higher in the ARF period than in the pre-ARF period, and in non-survivors compared to survivors (Figure 1B).

In the ARF period Pto/Cro significantly correlated in a negative manner with platelet counts (Figure 1C) and in a positive manner with white blood cell (WBC) counts (Figure 1D). By contrast, in the pre-ARF period no significant correlations were observed between Pto/Cro and platelet (y = 1.9501 + 0.00125, r 0.10041, p 0.27312) or WBC counts (y = 1.8256 + 0.00004, r 0.10481, p 0.25258).

### Proteinuria correlated with loss of glomerular and tubular functions

**Figure 2:**
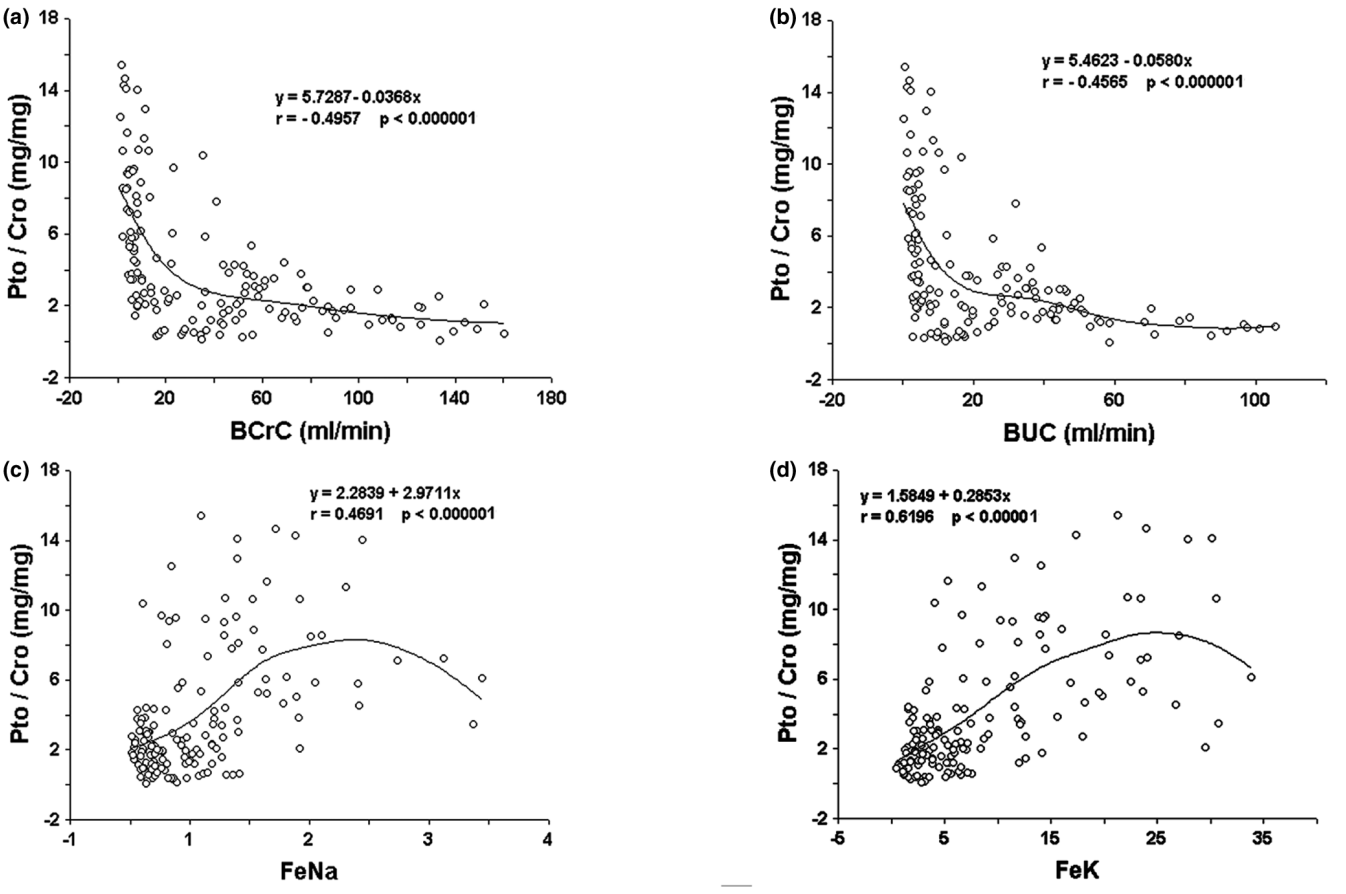
Correlation among proteinuria and indexes of glomerular and tubular function in acute renal failure (ARF) period. **(a, b) **Significant negative relationship between proteinuria/creatininuria ratio (Pto/Cro, mg/mg) and blood creatinine clearance (BCrC, ml/min, **(a)**) and blood urea clearance (BUC, ml/min, **(b)**). **(c, d) **Significant positive relationship between Pto/Cro and fractional excretion of sodium (FeNa, **(c)**) and potassium (FeK, **(d)**). A statistical linear regression test analysis was performed. Data for different parameters are also shown as minimal square fitting curves.

During the ARF period, Pto/Cro negatively correlated both with decreased BCrC and BUC (Figure 2A,B) and positively correlated both with FeNa and FeK (Figure 2C,D). The best linear correlation was observed between Pto/Cro and FeK, with a Pearson coefficient > 0.6 (Figure 2D). By contrast, in the pre-ARF period no significant correlations were observed between Pto/Cro and BCrC, BUC, FeNa and FeK.

### Burns septic ARF group plasma exerted a cytotoxic effect on tubular cells and on podocytes

**Figure 3:**
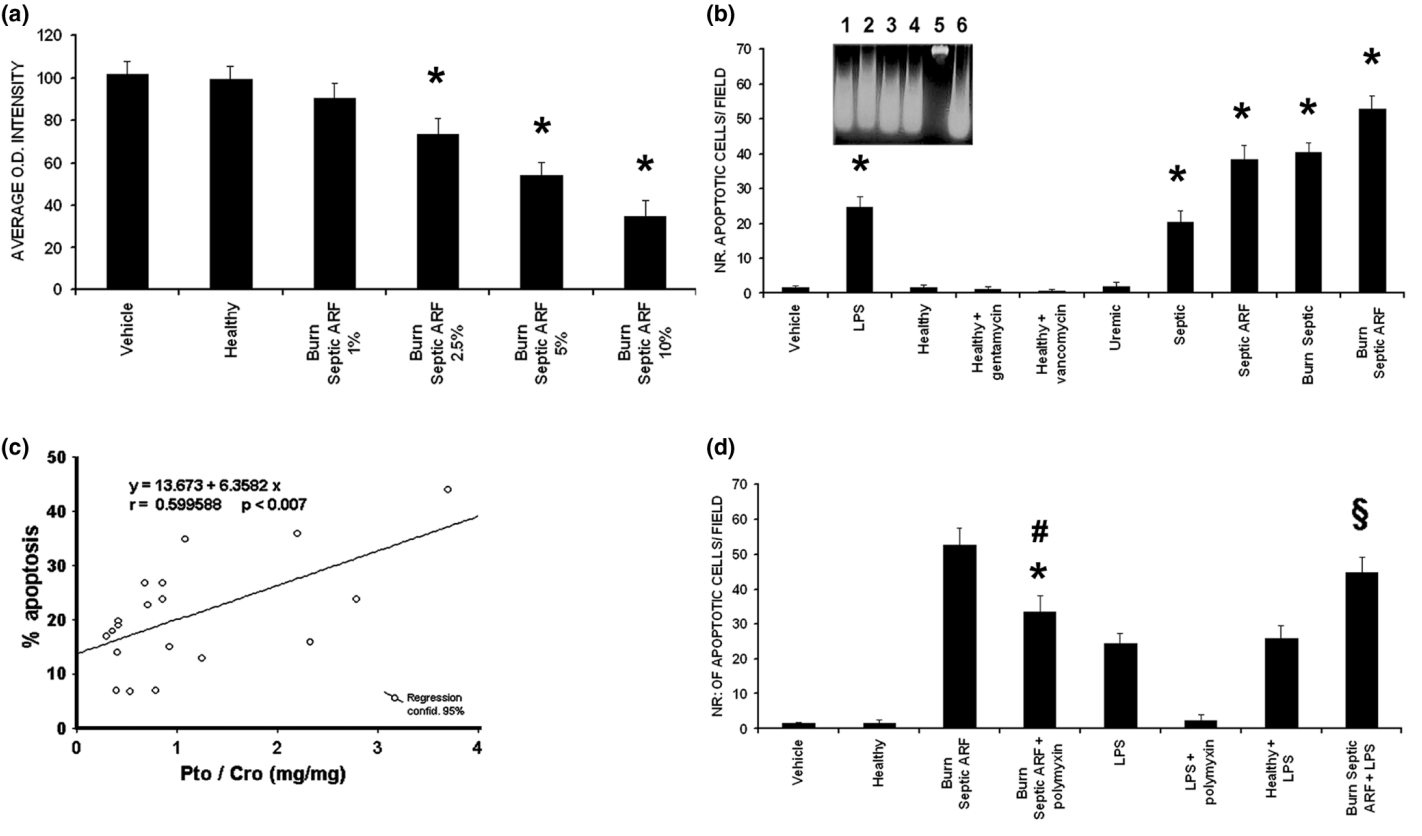
Pro-apoptotic effect of burns septic acute renal failure (ARF) group plasma on tubular cells and correlation with proteinuria. **(a) **Plasma from burns septic ARF group patients induced a dose-dependent decrease of tubular cell viability (XTT-based assay, n = 19, *p < 0.05 burns septic ARF group plasma 2.5%, 5% or 10% vs control healthy plasma). **(b) **Burns septic ARF group plasma (5%, 48 h of incubation, n = 19) induced a significant increase in tubular apoptosis (terminal uridine deoxynucleotidyl transferase dUTP nick-end labeling (TUNEL) assay, *p < 0.05 burns septic ARF group plasma vs control healthy plasma, healthy plasma + gentamicin, healthy plasma + vancomycin or uremic plasma). Gentamicin (2 μg/ml) or vancomycin (10 μg/ml) was added to control healthy plasma in selected experiments (n = 10). A significant increase of tubular apoptosis with a maximal effect with burns septic ARF group plasma (*p < 0.05 septic, septic ARF or burns septic vs all controls) was observed. LPS (30 ng/ml) was used as positive experimental control. **(b) **inset, typical DNA fragmentation of apoptotic tubular cells (burns septic ARF group plasma in lanes 1–4, positive control 30 ng/ml LPS in lane 6, negative control healthy plasma in lane 5). **(c) **Correlation between the percentage of tubular apoptosis induced by burns septic ARF group plasma (TUNEL assay) and Pto/Cro of the enrolled patients (n = 19). **(d) **Significant reduction of tubular apoptosis (TUNEL assay) after addition of 5 μg/ml polymyxin B (^#^p < 0.05 burns septic ARF group + polymyxin vs burns septic ARF group, n = 19). Lipopolysaccharide (LPS; 30 ng/ml) was used as internal control. Polymyxin B pre-treatment did not completely suppress plasma-induced apoptosis (*p < 0.05 burns septic ARF group + polymyxin vs control healthy plasma). Burns septic ARF group plasma but not control healthy plasma enhanced LPS-induced tubular apoptosis (^§^p < 0.05 burns septic ARF group plasma + LPS vs control healthy plasma + LPS). Values in **(a)**, **(b) **and **(d) **are expressed as averages ± standard error (SE). Each plasma was tested in triplicate. Analysis of variance (ANOVA) with Newman-Keuls multi-comparison test was performed. Linear regression analysis was performed in **(c)**.

Incubation with increasing doses of burns septic ARF group plasma (range 1–10%) induced a marked reduction of viability of tubular cells (Figure 3A) and of podocytes (data not shown). The cytotoxic effect was evident from 12 h, increased over time and peaked at 48–72 h (data not shown). No significant cytotoxic effect was observed after addition of control healthy group plasma.

### Burns septic ARF group plasma induced tubular cell apoptosis correlated with proteinuria

Consistent with the cytotoxicity data, we observed a significant increase of apoptotic tubular cells detected by TUNEL assay after incubation with lipopolysaccharide (LPS) (positive control), septic, septic ARF, burns septic and burns septic ARF group plasma (Figure 3B). The maximal effect was observed with burns septic ARF group plasma (Figure 3B), and a similar pro-apoptotic effect was also observed on podocytes (data not shown). Apoptosis was confirmed by detection of DNA fragmentation (Figure 3B, inset). Since burns patients were treated with potentially nephrotoxic antibiotics, we used healthy plasma as control in absence or presence of the same concentrations of vancomycin (10 μg/ml) and gentamicin (2 μg/ml) detected in the plasma of treated patients. Addition of antibiotics did not induce a significant increase of tubular apoptosis (Figure 3B). Furthermore, when we tested plasma from stable chronic uremic patients, no significant effects were observed (Figure 3B). The rate of tubular apoptosis induced by burns septic ARF group plasma significantly correlated with Pto/Cro ratio (Figure 3C).

The apoptotic effect of burns septic ARF group plasma could be only partially accounted to the presence of LPS. Indeed, pre-treatment of plasma with polymyxin B significantly reduced, but did not suppress this effect (Figure 3D), suggesting the presence in plasma of harmful mediator/s other than LPS. Indeed, the level of TNF-α (81.3 ± 9.4 pg/ml) in these plasma samples was significantly higher than in healthy controls (5.9 ± 0.8 pg/ml). In addition, pre-incubation of tubular cells with burns septic ARF group plasma and subsequent addition of LPS resulted in a significant worsening of tubular apoptosis in comparison to stimulation with LPS alone (Figure 3D).

**Figure 4:**
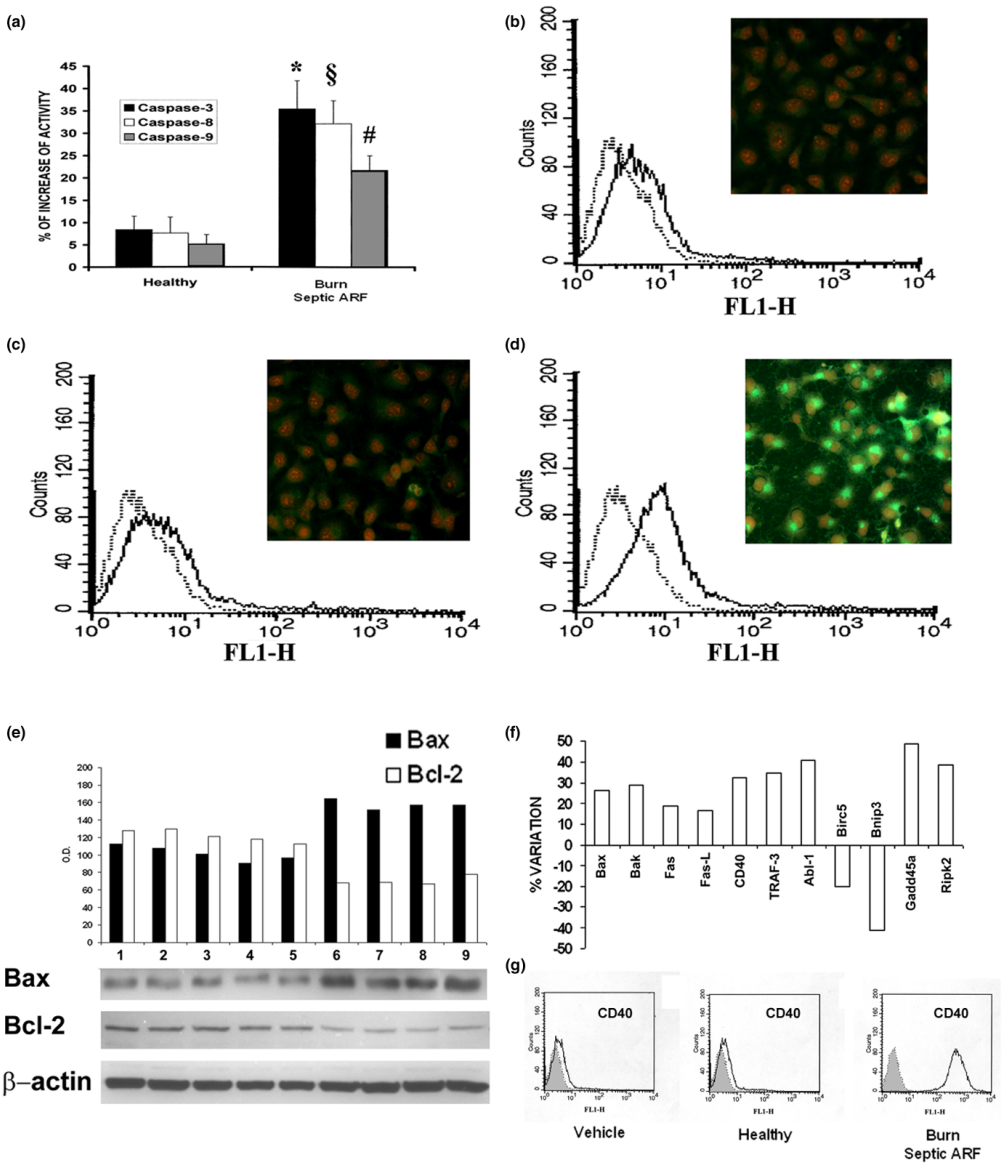
Burns septic ARF group plasma activated caspases, up-regulated Fas/CD40 and modulated Bax/Bcl-2 in tubular cells. **(a) **Significant increased activities of caspases 3, 8 and 9 on tubular cells incubated for 48 h with burns septic acute renal failure (ARF) group plasma (n = 19) in comparison to control healthy plasma (n = 10). All plasma samples were tested in triplicate. Student t test was performed: (p < 0.05 *caspase-3, ^§^caspase-8 and ^#^caspase-9 activities of burns septic ARF group vs control healthy plasma). **(b-d) **Representative images of fluorescence-activated cell sorting (FACS) and immunofluorescence (insets) analysis of Fas (CD95) expression on tubular cell surface after exposure to different stimuli. With respect to vehicle alone **(b) **or control healthy plasma **(c)**, burns septic ARF group plasma induced a marked up-regulation of Fas **(d) **(magnification × 400, nuclei counterstained with 1 μg/ml propidium iodide). Similar results were obtained with all tested plasma. **(e) **Up-regulation of the pro-apoptotic protein Bax and down-regulation of the antiapoptotic protein Bcl-2 in representative Western blot analysis on lysates of tubular cells (vehicle alone in lane 1, control healthy plasma in lanes 2–5, burns septic ARF group plasma in lanes 6–9) and related densitometric analysis. Beta-actin was used as reference for protein loading. **(f) **Percentage variation of expression of genes involved in apoptosis of tubular cells exposed to burns septic ARF group plasma. Tubular cells showed an increased expression of genes related to receptor-mediated (Fas, Fas-Ligand) and mitochondrial apoptotic pathways (Bax, Bak), of the co-stimulatory molecule CD40, of the CD40-transducer protein TRAF-3 and of the positive regulator of nuclear factor (NF)-κB activator RIPK2. Results are given as ratio between densitometric analyses of gene expression in tubular cells exposed to burns septic ARF group plasma with respect to control healthy plasma. House-keeping genes (beta-actin, GAPDH) were used as reference for densitometric analysis. Three experiments were performed with similar results. These data are feely available from the ArrayExpress databank of the European Bioinformatics Institute (see Materials and methods). Representative FACS analysis of CD40 expression was performed on unstimulated tubular cells (Vehicle), or in the presence of control healthy plasma, or burns septic ARF group plasma. Similar results were obtained with all tested burns septic ARF group plasma. The grey areas show the isotypic control.

The activity of caspases 3, 8 and 9 significantly increased in tubular cells after incubation with burns septic ARF group plasma (Figure 4A). Via FACS (Figure 4B–D) and immunofluorescence analysis (Figure 4B–D, insets), we observed a slight basal expression of Fas (Figure 4B). Fas expression did not change in the presence of healthy plasma (Figure 4C), whereas it was markedly up-regulated with burns septic ARF group plasma (Figure 4D).

In addition, burns septic ARF group plasma, but not control healthy plasma, induced the up-regulation of Bax and the down-regulation of Bcl-2 (Figure 4E), proteins known to modulate the mitochondrial apoptotic pathway. Gene array analysis demonstrated that burns septic ARF group plasma induced an up-regulation of several pro-apoptotic genes such as Fas, Fas-Ligand (Fas-L), Bax and Bak and of positive regulators of apoptosis (Abl1, Gadd45a). The negative regulators of apoptosis, Birc5 and Bnip3, were down-regulated. In addition, the CD40 gene and its transduction factor TRAF-3, as well as the nuclear factor (NF)-kB activator Ripk2 gene, were up-regulated, suggesting an inflammatory activation of tubular cells (Figure 4F). Up-regulation of CD40 was confirmed by FACS analysis (Figure 4G). This is consistent with previous observations indicating that CD40 is over-expressed in renal tubular epithelial cells during inflammatory injury [29].

### Burns septic ARF group plasma altered cytoskeleton distribution and megalin expression in tubular cells

**Figure 5:**
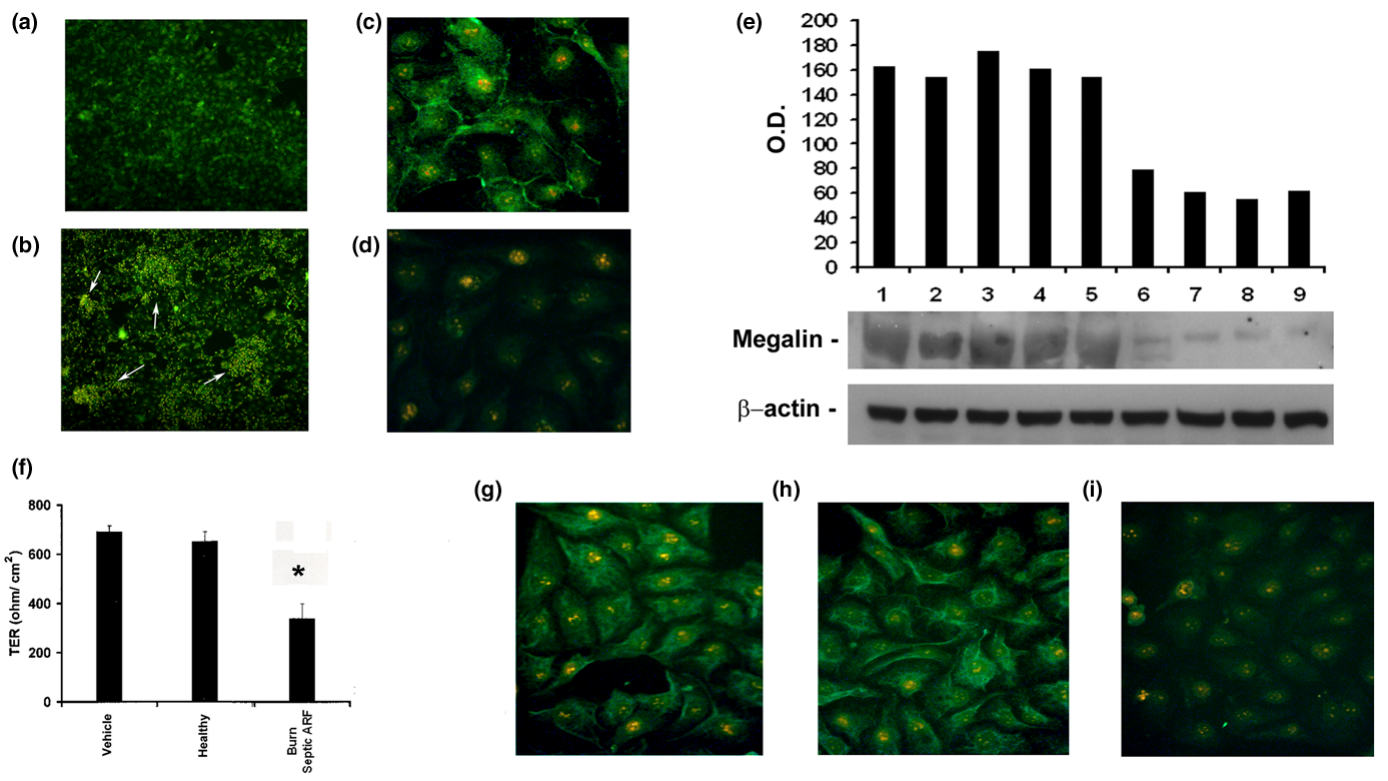
Burns septic acute renal failure (ARF) group plasma altered cytoskeleton, megalin and tight junction expression and polarity in tubular cells. **(a, b) **Representative micrographs showing normal distribution (**(a) **control healthy plasma) and marked re-arrangement of cytoskeleton actin on tubular cells exposed for 48 h to burns septic ARF group plasma **(b) **with formation of "heaps" (white arrows) visible via ultraviolet (UV) light microscopy after staining with fluorescein isothiocyanate (FITC)-conjugated phalloidin (magnification × 100). **(c, d) **Representative immunofluorescence of megalin expression on tubular cells incubated with control healthy plasma **(c) **and its down-regulation in presence of burns septic ARF group plasma **(d) **(magnification × 400, nuclei counterstained with 1 μg/ml propidium iodide). Similar results were observed with all tested plasma samples. **(e) **Representative Western blot analysis of megalin expression on tubular cells (vehicle alone in lane 1, control healthy plasma in lanes 2–5, burns septic ARF group plasma in lanes 6–9) and densitometric analysis. Beta-actin was used as reference for protein loading. **(f) **Significant loss of polarity of tubular cells evaluated by the decrease of trans-epithelial resistance (TER) normalized for the membrane area after incubation with burns septic ARF group plasma for 12 h (*p < 0.05 burns septic ARF group vs control healthy plasma). **(g-i) **Representative micrographs showing the expression of the tight junction protein ZO-1 on unstimulated tubular cells **(g)**, in the presence of control healthy plasma **(h) **and its down-regulation after incubation with burns septic ARF group plasma **(i) **(magnification × 400, nuclei counterstained with 1 μg/ml propidium iodide). Similar results were obtained with all tested plasma samples. Values in **(f) **are expressed as averages ± standard error (SE). Each plasma sample was tested in triplicate. Analysis of variance (ANOVA) with Newman-Keuls multi-comparison test was performed.

Burns septic ARF group plasma induced a marked alteration of distribution of cytoskeleton actin fibers in tubular cells. In comparison to control healthy plasma (Figure 5A), burns septic ARF group plasma promoted the formation of "heaps" (Figure 5B) composed of tubular cells grouped in clusters, an effect likewise ascribed to apoptosis.

We then tested whether burns septic ARF group plasma could induce early alterations in tubular cells not accountable to apoptosis such as down-regulation of megalin, an endocytic receptor involved in re-absorption of filtered proteins. Burns septic ARF group plasma provoked a marked down-regulation of megalin (Figure 5D–E). By contrast, control healthy plasma did not alter the normal expression pattern of megalin (Figure 5C–E).

### Burns septic ARF group plasma altered tubular cell polarity

The loss of polarity of tubular cells was studied by evaluating TER. After incubation with burns septic ARF group plasma, tubular cells exhibited significantly lower TER values in comparison to healthy controls (Figure 5F). Burns septic ARF group plasma also induced a decreased expression of E-cadherin and pan-cytokeratin epithelial markers (data not shown) and of the tight junction protein ZO-1 (Figure 5G–I).

### Burns septic ARF group plasma altered polarity, cytoskeleton distribution, permeability and nephrin expression in podocytes

**Figure 6:**
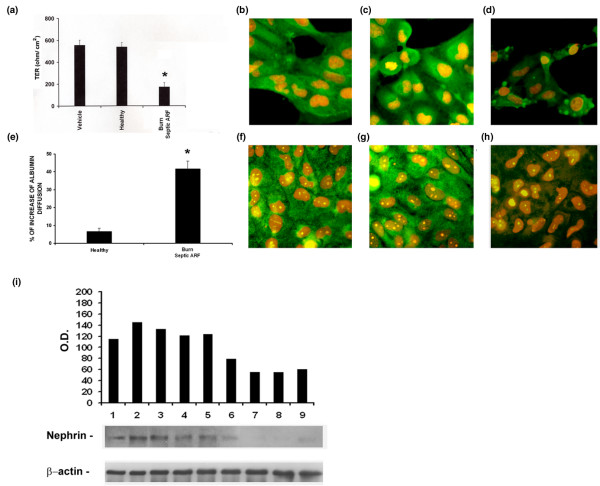
Burns septic acute renal failure (ARF) group plasma altered polarity, permeability to albumin and nephrin expression on podocytes. **(a) **Significant variation of trans-epithelial resistance (TER) after exposure to burns septic ARF group plasma (*p < 0.05 burns septic ARF group vs control healthy plasma). **(b-d) **Representative immunofluorescence images of the altered distribution of the intermediate filament protein nestin in presence of burns septic plasma **(d)**, not detectable after incubation with vehicle alone **(b) **or control healthy plasma **(c) **(magnification × 400, nuclei counterstained with 1 μg/ml propidium iodide). After burns septic plasma challenge, nestin lost its typical diffuse distribution and was localized in the sub-membrane spaces forming several "rings" **(d)**. **(e) **Significant increased diffusion of albumin across podocyte monolayers exposed to burns septic ARF group plasma (*p < 0.05 burns septic plasma vs control healthy plasma). **(f-h) **Representative immunofluorescence images of the slit diaphragm protein nephrin after exposure to vehicle alone **(f)**, control healthy plasma **(g) **and burns septic ARF group plasma **(h)**. Burns septic ARF group plasma induced a diffuse loss of nephrin (magnification × 400, nuclei counterstained with 1 μg/ml propidium iodide). **(i) **Representative Western blot analysis of podocyte nephrin expression (vehicle alone in lane 1, control healthy plasma in lanes 2–5, burns septic ARF group plasma in lanes 6–9) and related densitometric analysis. Beta-actin was used as reference for protein loading. Values in **(a) **and **(e) **are expressed as averages ± standard error (SE). Each plasma was tested in triplicate. Analysis of variance (ANOVA) with Newman-Keuls multi-comparison test was performed.

After incubation with burns septic ARF group plasma, podocytes exhibited significantly lower TER values in comparison with healthy controls (Figure 6A). Burns septic ARF group plasma also induced a marked redistribution of actin fibers (data not shown) and of the intermediate filament protein nestin (Figure 6B–D). Nestin filaments appeared concentrated under the plasma membrane (Figure 6D), when compared with the diffuse cellular staining of controls (Figure 6B–C).

Burns septic ARF group plasma led to a significant average increase of albumin diffusion in comparison to healthy controls (Figure 6E). The expression of nephrin, a slit diaphragm protein, whose alteration is deeply involved in the pathogenic mechanisms of proteinuria, was also investigated. In the presence of vehicle alone (Figure 6F) or control healthy plasma (Figure 6G), podocytes showed a positive diffuse staining for nephrin that was almost completely abrogated after incubation with burns septic ARF group plasma (Figure 6H). These results were confirmed by Western blot analysis (Figure 6I).

## Discussion

In the present study we demonstrate that plasma from patients with severe burns and sepsis-associated ARF contains factors that induce functional alterations in tubular cells and podocytes. Plasma-induced apoptosis on tubular cells correlated with the extent of proteinuria, which in turn was related to the severity of the septic process, with impairment of renal function, and with patient outcome.

We found that proteinuria, expressed as Pto/Cro ratio in order to avoid the tubular concentration factor, progressively increased after the onset of ARF and was higher in non-survivors than in survivors, as recently reported [30]. We also observed that proteinuria in the ARF period was inversely correlated with BCrC and BUC and directly with FeNa and FeK. These correlations suggest that, at least in this group of patients, proteinuria is a reliable index of the severity of glomerular and tubular injury.

In the ARF period, proteinuria significantly correlated with platelet and WBC counts, which are activated during sepsis [31-33].

In burns patients with sepsis and ARF, the mechanisms of late and sustained glomerular and tubular injury are complex, involving hemodynamic changes, tissue breakdown products, development of septicemia and drug nephrotoxicity [10-13].

In the present study, we found that sepsis was a determinant factor for the induction of apoptotic injury of kidney cells. Indeed, plasma from burns patients with sepsis-associated ARF exerted an enhanced pro-apoptotic effect on tubular cells, suggesting that burns and ARF could be linked in worsening sepsis-induced apoptosis [4-6,34]. The apoptotic activity of plasma was also found to correlate with Pto/Cro.

Previous studies suggested a link among sepsis, plasma concentrations of pro-apoptotic molecules and kidney injury. *In vitro*, LPS and inflammatory cytokines promoted tubular cell apoptosis by up-regulating Fas expression and caspase activity [9]. In clinical studies in burns patients, plasma soluble Fas levels were found to be significantly higher in non-survivors than in survivors [35].

Here, we show that burns septic ARF group plasma induced both activation of caspases and up-regulation of Fas on tubular cells. Our data suggest an involvement of both Fas death-receptor and mitochondrial tubular apoptotic pathways. Indeed, we observed a down-regulation of the anti-apoptotic protein Bcl-2 and an up-regulation of the pro-apoptotic protein Bax. These results are consistent with data observed in experimental animals injected with LPS [36-38].

Moreover, burns septic ARF group plasma significantly enhanced tubular apoptosis induced by LPS. In these patients, plasmatic peaks of LPS can induce direct renal damage and can also stimulate further production of inflammatory mediators [5-9]. We found that the effect of burns septic ARF group plasma on tubular apoptosis was partially inhibited, but not suppressed, by LPS blockade, suggesting that mediators other than LPS contributed to such phenomenon. In our experimental setting, we found enhanced levels of TNFα, which is known to induce tubular apoptosis. Moreover, the high serum levels of a broad range of cytokines detected in these patients [34] suggest the involvement of multiple mediators in specific organ injury [8,9,39].

In the first weeks after burn injury, proteinuria is usually in the nephrotic range with a mixed glomerular and tubular pattern [13], suggesting a simultaneous defect of tubular re-absorption and an increase of glomerular permeability. Megalin is an endocytic receptor that plays a pivotal role in the normal re-absorption of filtered proteins [40]. Burns septic ARF group plasma induced a significant decrease of megalin expression on tubular cells, suggesting that the impairment of tubular re-adsorption due either to apoptosis or to loss of megalin expression could contribute to proteinuria, resulting in the failure of tubular handling of filtered proteins.

We also observed a marked down-regulation of nephrin expression in cultured podocytes after exposure to burns septic ARF group plasma. Nephrin is a protein of the slit diaphragm of podocytes that regulates glomerular permeability. Its redistribution and loss has been shown to occur in patients with congenital or acquired nephrotic syndrome [41,42]. In addition, we observed a redistribution of actin cytoskeleton fibers and of the intermediate filament protein nestin. Nestin is able to stably link the intermediate filaments to other cytoskeleton proteins, playing a role in the correct organization and function of podocytes [43]. The alterations in nephrin and cytoskeleton distribution might also account for the altered cell polarity and albumin transport across the podocyte monolayer observed after challenge with burns septic ARF group plasma.

In this clinical setting, several other tubular abnormalities including increased fractional sodium excretion and uric acid clearance, low threshold of phosphate adsorption, glycosuria and aminoaciduria have been described [11-13,20]. We found a significant correlation between proteinuria and urine loss of sodium (r = 0.46) and potassium (r = 0.60). Physiological tubular handling of electrolytes is based on the maintenance of cell polarity and on the integrity of tight junction protein expression [44]. After challenge of tubular cells with burns septic ARF group plasma, we observed a marked decrease of ZO-1 expression with a simultaneous alteration of TER. These functional changes could alter the ability of tubular cells in maintaining compositionally distinct fluid-filled compartments with precise electrolyte concentrations.

## Conclusion

The results of the present study demonstrate that plasma samples from septic patients with severe burns contain pro-apoptotic factors that could contribute to the development of renal injury and proteinuria. These findings provide a further rationale for ongoing clinical studies aimed at the use of drugs that inhibit apoptosis [45] and therapeutic removal of inflammatory mediators by extracorporeal techniques [46]. Strategies aimed at increasing the removal of cytokines from the circulation could prevent the onset of ARF and improve the clinical outcome for these patients [46].

## Key messages

Renal dysfunctions and proteinuria are constant clinical findings in septic severe burns patients.

Proteinuria is correlated to outcome, glomerular and tubular function impairment and systemic inflammation indexes.

Plasma from septic burns patients induced a pro-apoptotic effect that correlated with the extent of proteinuria in tubular cells and podocytes.

Plasma from septic burns patients also exerted an alteration of tubular cell polarity, a reduced expression of the tight junction protein ZO-1 and of the endocytic receptor megalin.

In podocytes, burns septic group plasma increased permeability to albumin and decreased the expression of the slit diaphragm protein nephrin.

## Abbreviations

ADQI = Acute Dialysis Quality Initiative; ARF = acute renal failure; BCrC = blood creatinine clerance; BUC = blood urea clearance; FeNa = fractional sodium excretion; FeK = fractional potassium excretion; LPS = lipopolysaccharide; Pto/Cro = proteinuria/creatininuria ratio; RRT = renal replacement therapy; TER = trans-epithelial resistance; WBC = white blood cells.

## Competing interests

The authors declare they have no competing interests.

## Authors' contributions

FM and VC conceived the study, analyzed and interpreted the data and elaborated the manuscript. FM, MS and MC performed the enrollment of patients in the study and carried out the analysis of clinical and biochemical parameters. VC, GMR and BA performed *in vitro *studies on tubular cells and glomerular podocytes. LB, GT and MR participated in the design of the study and interpreted clinical and laboratory results. GC conceived and supervised the study, analyzed and interpreted the data and corrected the final version of the manuscript. All authors read and approved the final version of the manuscript.
